# Summer temperature and all-cause mortality from 2006 to 2015 for Hyderabad, India

**DOI:** 10.4314/ahs.v21i3.59

**Published:** 2021-09

**Authors:** Suresh K Rathi, Prahlad R Sodani

**Affiliations:** 1 IIHMR University, Jaipur & MAMTA Health Institute for Mother and Child, New Delhi - 110048. rathisj07@gmail.com; 2 President, IIHMR University 1, Prabhu Dayal Marg, Near Sanganer Airport, Jaipur - 302029. sodani@iihmr.edu.in

**Keywords:** Heat wave, all-cause mortality, urban, humidity, heat index, India

## Abstract

**Background:**

Studies have documented a significant association between temperature and all-cause mortality for various cities but such data are unavailable for Hyderabad City.

**Objective:**

The objective of this work was to assess the association between the extreme heat and all-cause mortality for summer months (March to June) from 2006 to 2015 for Hyderabad city population.

**Methods:**

We obtained the data on temperature and all-cause mortality for at least ten years for summer months. Descriptive and Bivariate analysis were conducted. Pearson correlation coefficient was used to study the relationship between heat and all-cause mortality for lag time effect.

**Results:**

A total of 122,117 deaths for 1,220 summer days (2006 to 2015) were analyzed with mean daily all-cause mortality was 100.1±21.5. There is an increase of 16% and 17% per day mean all-cause mortality at the maximum temperature of ≥40°C and for extreme danger days (Heat Index >54°C) respectively. The mean daily all-cause mortality shows a significant association with maximum temperature (P < 0.001) and Heat Index from caution to extreme danger risk days (P < 0.0183). The lag effect of extreme heat on all-cause mortality for the study period (2006 to 2015) was at peak on same day of the maximum temperature (r = 0.273 at p<0.01).

**Conclusion:**

The study concludes that the impact of ambient heat in the rise of all-cause mortality is clearly evident (16% mean deaths/day). There was no lag effect from the effect of extreme heat on all-cause mortality as the peak period was the same as the maximum temperature. Hence heat action plans are needed. However, extreme heat-related mortality merits further analysis.

## Introduction

Global warming and the El Nino events in 2015 and 2016 resulted in high temperatures across the planet 1, 2. The WHO estimates that between 2030 and 2050 global climate change is predicted to cause a further 250,000 deaths per annum mainly associated with malaria, malnutrition, diarrhoea and heat stress 3. Rising temperature is nearly the universal phenomena and scientists are researching and reporting its impact on health, development and productivity. Chronic exposure to extend or changes in heat and humidity (including and beyond episodic heatwaves) results in impacts on behavioural, physical and psychological state and mortality 4. Impact is often amplified in urban areas and may cause impacts on labour and overall productivity, with associated economic aftermath as urban areas may face higher levels of temperature than adjacent suburb and rural areas due to the Urban Heat Island (UHI) effect 5. The mortality impact of high heat has been explored for several regions of the planet 6,7,8. India also witnessed a series of heat waves with considerable mortality 9,10,11. The destructive impact of one wave in India in May 2015, with over 2200 fatalities, demonstrated that extreme heat may be a serious issue even in countries regularly exposed to high temperatures 12. Heatwaves are expected to increase further not only in intensity, but also in duration and frequency 11, 13, 14.

Generally, the linkages between heat and all-cause mortality are studied at broader scales which are less likely relevant at local (city) level. Coastal, desert and dry cities could also be different from global climate change perspective and its mitigation measures. Therefore, generation of local evidence during this domain is critical to first understand the character and magnitude of health effects, then to develop and implement plans for risk mitigation.

To the authors knowledge there have been no studies in the Hyderabad area with Telangana reporting high number of heat stroke cases and deaths due to heat waves^15^, ^16^. Hyderabad is a very urbanized city in Telangana with high heat wave risk according to news reports. Hyderabad has been selected for robust but scientific evidence generation which may be helpful in the planning of heat wave mitigation efforts. The aim of this paper is to estimate the impact of heat wave on mortality in Hyderabad. It is important to review the cumulative deaths at the town level. The target of the study is to characterize the acute heat (daily maximum temperature, daily minimum temperature, daily mean temperature, and Heat index (HI) impacts on all-cause mortality for summer months (March to June) from 2006 to 2015 for urban population of Hyderabad.

## Methods

### Study Area

Hyderabad is Telangana's capital and largest Indian state city, covering 650 square kilometers along Musi River banks. Hyderabad's population is 6.8 million (2011 Census) with a population density of 10,477 people per square kilometer making it the fourth-populated Indian city. Hyderabad has a dry and humid tropical climate bordering on a hot semi-arid climate. The mean annual temperature is 26.6°C; average monthly temperatures range from 21–33°C. Summers (March – June) are hot and humid, with mean highs in the mid-to-high 30s Celsius; between April and June, maximum temperatures often exceed 40°C. May is the hottest month, as temperatures range from 26°C to 39°C each day. Between June and September, heavy rain from the southwest summer monsoon comes providing Hyderabad with much of its mean annual rainfall.

### Study Design

Retrospective cross sectional analysis of all-cause mortality data with temperature, humidity and heat index for Hyderabad city.

### Data Collection

i) Climatic data (Temperature and Humidity Data)

The data (secondary) on Temperature and Humidity were collected through online from Tutiempo Network, S.L http://www.tutiempo.net/en/Climate/.html^17^ for the summer months for the years of 2006 to 2015. The values were collected for daily average, maximum and minimum temperature (°C) and daily average relative humidity (%) for Hyderabad City. Daily maximum temperature and relative humidity data were used in the calculation of the HI.

**Summer Day:** It is defined as any day from March to June.

**Heat Index:** HI (feel like temperature) is a measure to estimate how hot it really feels when relative humidity is factored with the actual air temperature. Rothfusz equation 18 for HI calculation is applied as it is widely in practice for cities 5, 11, 19, 20.

Heat Index equation:

Where, T = ambient dry bulb temperature (°C) and R = relative humidity (%).

The HI was further classified into Extreme Danger: >54°C; Danger: 41°C - 54°C; Extreme Caution: 32°C – 41°C and Caution: 27°C – 32°C 11.

**ii) Mortality Data**

The mortality data (secondary) of the Hyderabad city were obtained from the Birth and Death Registration Department of the Municipal Corporation / Municipality for the years from 2006 to 2015 through website (http://www.ghmc.gov.in/deaths/deathssearch.asp?id=1&circlepd=&circle=1&ward=&date1=01-Jan-2016&date2=14-Jun-2016&dname=&dsex=A&offset=90).

### Ethical Clearance

The study has utilized existing (secondary) data, and therefore, no direct interaction was made with human beings. The data do not have any identifying information (anonymous in analysis). However, ethical clearance has been taken from the Institutional Committee for Ethics and Review of Research of IIHMR University.

### Statistical Analysis

Data were processed in Microsoft Excel and analyzed through Stata (14.2) and SPSS (20.0). Descriptive, and bivariate analysis were conducted. Numerical data were expressed as the means and standard deviation (means ± s.d.). The student's t-test and ANOVA were used to examine differences in the means of variables (average temperature, maximum temperature, and heat index). ANOVA was used for testing the significance of differences between the mean all-cause mortality (death) values at maximum temperature of <35°C, 35–39°C and ≥40°C and heat index of 27–32°C, 32–41°C, 41–54°C and >54°C. Pearson correlation analysis was used to determine the relationship for the maximum temperature, average temperature, minimum temperature, relative humidity and heat index with all-cause mortality for lag time effect. The probability (p) level of ≤0.05 was considered significant.

## Results

A total of 122,117 deaths (all-cause mortality) for 1,220 summer days (2006–2015) were analyzed with temperature and relative humidity.

Table-1 shows that mean maximum temperature, mean relative humidity and mean heat index during summer were 37.8 ± 3.5°C, 41.1 ± 14.7% and 43.3 ± 5.3°C respectively for the study period. The yearly mean maximum temperature during summer ranges from 36.2 ± 3.4°C for the year 2006 to 38.8 ± 3.2°C in 2010. The highest recorded value for maximum temperature, relative humidity and heat index during summer were 46.0°C, 95% and 68.9°C respectively for the study period. The yearly mean HI is lowest in 2009 (41.7 ± 4.1°C) and highest in 2012 (44.7±5.5°C).

**Table 1:** Year-wise statistics of mean and range of maximum temperature, average relative humidity, heat index (HI) and days with maximum temperature ≥40°C for the years 2006 to 2015, Hyderabad

Year	Maximum Temperature °C (mean ± SD)	Range of Maximum Temperature (°C)	Average Relative Humidity (%)	Highest recorded value of Relative Humidity (%)	HI (°C) (mean ± SD)	Highest recorded value of HI (°C)
2006	36.2 ± 3.4	26.7 – 42.0	51.5 ± 12.9	85	44.1 ± 5.1	58.1
2007	37.2 ± 3.6	27.0 – 42.5	45.2 ± 14.6	85	43.6 ± 5.7	56.4
2008	36.6 ± 3.4	23.2 – 41.9	44.5 ± 15.2	95	41.9 ± 3.9	51.6
2009	38.4 ± 2.7	29.4 – 43.4	33.9 ± 11.0	64	41.7 ± 4.1	50.7
2010	38.8 ± 3.2	30.1 – 44.5	38.3 ± 14.8	81	44.3 ± 4.9	58.8
2011	37.9 ± 2.5	30.0 – 42.1	36.9 ± 11.5	63	42.3 ± 5.1	59.4
2012	38.8 ± 3.0	29.1 – 43.1	38.7 ± 13.3	81	44.7 ± 5.5	68.9
2013	38.5 ± 4.2	27.0 – 46.0	38.4 ± 16.9	80	43.5 ± 5.8	56.4
2014	38.3 ± 3.7	27.0 – 44.0	39.2 ± 12.9	82	43.5 ± 5.9	61.6
2015	37.1 ± 3.7	28.8 – 45.0	44.4 ± 14.7	84	43.3 ± 6.0	61.8
2006–2015	37.8 ± 3.5	23.2 – 46.0	41.1 ±14.7	95.0	43.3 ± 5.3	68.9

Table-2 shows that the mean daily all-cause mortality has been estimated at 100.1±21.5 for the study period. The minimum and maximum mean daily mortality is 79.3 ± 10.3 and 126.7 ± 15.1 for the year 2010 and 2009 respectively. The year 2013 was warmer in terms of high temperature with the maximum temperature of ≥40°C for 57 days as compared to the rest of the study period.

**Table 2:** Year-wise summer days, highest recorded maximum temperature and all-cause mortality from 2006 to 2015, Hyderabad

Year	No. Of Summer Days	No. Of Days with Maximum Temperature ≥40°C	Summer All-cause Mortality (Total Deaths)	Mean All-cause Mortality (per day)
2006	122	20	11,606	95.13 ± 13.32
2007	122	32	12,167	99.72 ± 15.06
2008	122	32	11,728	96.13 ± 13.98
2009	122	38	15,459	126.71 ± 15.07
2010	122	50	9,670	79.26 ± 10.33
2011	122	31	15,112	123.86 ± 11.92
2012	122	46	14,257	116.86 ± 20.18
2013	122	57	10,167	83.33 ± 13.21
2014	122	55	9,986	81.85 ± 10.46
2015	122	29	11,965	98.07 ± 14.73
2006–2015	1220	390	122,117	100.10 ± 21.5

The mean number of deaths (both for male and female) per day at maximum temperature of less than 35°C was 91.11 which increased to 105.71 at daily maximum temperature of 40°C and above. This shows that 14.6 (16%) mean deaths per day increased at the maximum temperature of 40°C and above. The mean number of deaths per day for less risky or caution days (HI: 27–31°C) was 87.33 which increased to 101.96 at HI of more than 54°C (extreme danger / most risky days). This shows that there was a per day rise of 14.63 (17 %) mean deaths for extreme danger level days (Table-3).

**Table-3:** All-cause mortality (Male, Female and Total) with summer temperature and HI for the years 2006 to 2015, Hyderabad City

Factor	Day	Male Mean deaths / Day (95%CI)	Female Mean deaths / Day (95% CI)	All-Cause Mortality Mean deaths / Day (95% CI)
**T Max (°C)**				
<35	231	58.47 (57.01–59.93)	32.64 (31.62–33.65)	91.11 (89.05–93.18)
35–39	599	63.71 (62.63–64.79)	36.18 (35.49–36.87)	99.89 (98.34–101.44)
≥40	390	66.50 (64.84–68.16)	39.21 (38.11–40.31)	105.71 (103.18–108.24)

**T Max (°C)**				
<45	1216	63.58 (62.77–64.39)	36.47 (35.93 – 37.01)	100.06 (98.85–101.26)
≥45	4	72.25 (48.49–96.00)	38.75 (23.85 – 53.64)	111.00 (74.55–147.44)

**Heat Index (°C)**				
27 – 32	15	55.00 (45.41–64.58)	32.33 (26.39–38.26)	87.33 (72.88–101.77)
32 – 41	397	62.97 (61.62–64.33)	35.37 (34.48–36.26)	98.35 (96.38–100.32)
41 – 54	774	64.04 (63.00–65.07)	37.11 (36.41–37.80)	101.15 (99.59–102.71)
>54	33	65.06 (59.76–70.35)	36.90 (33.41–40.40)	101.96 (94.61–109.32)

*121 Days for the year 2008 for HI

Table-4 depicts that the mean daily mortality shows a significant association with daily maximum temperature (P < 0.001) and HI or discomfort index from caution to danger risk days (P < 0.0183).

**Table 4:** Association of Temperature and HI with summer all-cause mortality for the years 2006 to 2015, Hyderabad City

Variable	Categories	n=1220	Mean deaths per day	t-test / F Test
Temperature (Average)°C	35–39	115	110.40	5.46 (p<0.001)
	<35	1105	99.02	

Temperature (Maximum)°C	<35	231	91.11	F = 35.41 (p<0.000)
	35–39	599	99.89	
	≥40	390	105.71	

Heat Index °C	27–32	15	87.33	F = 3.35 (p<0.0183)
	32–41	397	98.35	
	41–54	774	101.15	
	>54	33	101.96	

The lag effect of extreme heat on all-cause mortality for the study period (2006 to 2015) was at peak period on same day of the maximum temperature (r = 0.273 at p<0.01) but continues in decreasing order for next four days. While the lag effect of average temperature was high on same day (r = 0.228 at p<0.01) but for minimum temperature it was increasing for next two days (r = 0.134 at p<0.01) (Table-5).

**Table 5:** Lag time correlation of temperature and humidity with all-cause mortality counts (2006 to 2015), Hyderabad city

Pearson Correlation Coefficient	No lag	1 day	2 days	3 days	4 days
Average Temp (°C)	0.228**	0.209**	0.200**	0.181**	0.185**
Maximum Temp (°C)	0.273**	0.266**	0.259**	0.241**	0.226**
Minimum Temp (°C)	0.127**	0.131**	0.134**	0.121**	0.117**
Humidity (%)	-0.292**	-0.274**	-0.262**	-0.245**	-0.236**
HI (°C)	0.071*	0.079**	0.090**	0.085**	0.077**

* p<0.05

** p<0.01

## Discussion

Since 2010 onwards, heat waves in South Asia have been increasing especially in India 5. These heat waves have caught the attention of the public health experts, policy makers, and climatologists who wish to develop the early warning system, heat action plans and increase community awareness to improve preventive measures for heat waves 21. Due to climate change, extreme heat days are increasing which may lead to increase in all-cause mortality 22. High temperature, especially along side humidity may be a matter of concern for many of the cities / urban areas in reference to heat morbidity and mortality. This study investigated the association of daily maximum temperature, daily minimum temperature, humidity and Heat index with all-cause mortality in Hyderabad City. There is an observed association between heat, heat index and all-cause mortality for Hyderabad city. Although similar heat-mortality studies have been conducted in western countries and some studies are available from Southeast Asia including India, 5,6,7,8,9,10 but to the best of our knowledge, there are no studies available on the urban population of Hyderabad city.

There is a significant rise in the all-cause mortality risk with rise of maximum temperature consistent with other studies 23,24,25,26,27. Findings also show that moderate increase in maximum temperature may lead to large rises of all-cause mortality, a consistent with study by Mazdiyasni O, et.al. 28. Further, the findings substantiate that maximum temperatures between 35–39°C contributed to higher attributable risks of all-cause mortality than <35°C and ≥40°C maximum temperatures in India during 2006–2015, consistent with previous findings 29. We document a substantially greater number of deaths attributable to moderately hot temperature than to extremely hot temperatures. This is partly due to the higher proportion of moderately hot days than extremely hot during the study period.

Although the 2011 and 2015 had less number of days with temperature equal to or more than 40°C but mean daily deaths were higher for the year 2011 and almost similar for the year 2015 with reference to mean allcause mortality for the entire study period. This can be explained by the fact that temperature may be more high for those summer days which can result high allcause mortality.

The high heat index values seem to be well correlated with the all-cause mortality, this confirms Desai et al., and Monteiro et al. results 5, 30. Maximum temperature as well as heat index are correlated with increase in allcause mortality as evident from this analysis by increase of 16% deaths/day and 17% deaths/day. Hence, there is a need for the inclusion of humidity measures while calculating all-cause mortality impacts of ambient heat. The present analysis reveals that there were 390 out of 1,220 summer days having temperature ≥40°C and 807 out of 1,220 summer days having feel temperature /heat index ≥41°C. Both these are critical not just for increased all-cause mortality but also work performance of the population which may have an impact on the economy of Hyderabad, and personal health that may lead to dangerous heat disorders like muscle cramps, heat strokes 31. These findings support the efforts of National Disaster Management Authority 32 and various Non-governmental organizations to build up the resilience of these vulnerable populations to more severe heat waves. Hence, the authorities in Hyderabad needs to plan and implement interventions to such adverse climatic heat affects.

## Limitations

Few limitations of this analysis must be acknowledged.

1. Due to unavailability of data on age or cause of death, all-cause mortality data were analyzed as done previously 9,33.

2. This analysis was unable to isolate the heat related mortality due to unavailability of cause specific deaths. Hence we used all-cause mortality as an outcome variable and our estimate may be an overestimate or underestimate of the true effect of the heat and humidity on mortality.

3. Since retrospective data were used, its accuracy and completeness could not be fully verified.

## Conclusion

A total of 122,117 deaths (all-cause mortality) for 1,220 summer days (2006–2015) were analyzed in reference to temperature and relative humidity. The study concludes that the maximum temperature of ≥40°C and HI >41°C are the important predictors for all-cause mortality of urban population of Hyderabad. The mean daily allcause mortality has been estimated at 100.1±21.5 for the study period. There is an increase of 16% per day all-cause mortality at the maximum temperature of 40°C and above. Almost similar rise of 17% per day all-cause mortality for extreme danger days (HI>54°C). Mean daily all-cause mortality demonstrates an significant association with daily maximum temperature and HI. The effect of extreme heat on all-cause mortality for the study period (2006 to 2015) was at a peak on same day of the maximum temperature. Maximum temperature should be given more importance than average and minimum temperature while calculating the lag effect. Our findings contribute to a better understanding of local heat wave and hot days which can support the design for local thermal comfort standards and early warning systems for heat wave. Extreme heat related mortality merits further analysis in order to reduce harmful health effects among Hyderabad's most vulnerable population.

## Recommendations

The findings from this study demonstrate the need for preventive actions to protect the population from the effects of temperature at city level. While the Telangana government has developed a state heat action plan we believe that specific plans for urban populations be developed. Therefore Hyderabad city should develop a specific heat action plan. Further research can be directed on heat wave with age and cause-specific mortality. We also recommend that heat index should be considered while calculating all-cause mortality due to extreme heat.
